# Effect of* Xylocarpus granatum* Bark Extract on Amelioration of Hyperglycaemia and Oxidative Stress Associated Complications in STZ-Induced Diabetic Mice

**DOI:** 10.1155/2019/8493190

**Published:** 2019-05-02

**Authors:** Swagat Kumar Das, Arpita Prusty, Dibyajyoti Samantaray, Mojeer Hasan, Srikanta Jena, Jayanta Kumar Patra, Luna Samanta, Hrudayanath Thatoi

**Affiliations:** ^1^Department of Biotechnology, College of Engineering and Technology, Biju Patnaik University of Technology, Bhubaneswar, Odisha-751003, India; ^2^Redox Biology Laboratory, Department of Zoology, School of Life Sciences, Ravenshaw University, Cuttack, Odisha 753003, India; ^3^Microbial & Pharmaceutical Biotechnology Laboratory, Faculty of Pharmacy, Jamia Hamdard University, Hamdard Nagar, New Delhi 110062, India; ^4^Research Institute of Biotechnology & Medical Converged Science, Dongguk University-Seoul, Goyang-si, Republic of Korea; ^5^Department of Biotechnology, North Orissa University, Sriram Chandra Vihar, Takatpur, Baripada, Odisha 757003, India

## Abstract

*Xylocarpus granatum *is a medicinal mangrove plant, traditionally used for the treatment of diarrhoea, cholera, fever, dyslipidaemia, inflammation, etc. The present study was aimed to evaluate the* in vitro* antidiabetic (*α*-glucosidase inhibition assay) and antioxidant (ABTS scavenging and metal chelating assay) activities of ethanol, methanol, and aqueous extracts of leaves and barks of* X. granatum* followed by* in vivo* antidiabetic and antioxidant evaluation of ethanol bark extracts in streptozotocin- (STZ-) induced diabetic mice. The* in vitro* evaluation revealed higher *α*-amylase inhibition and ABTS scavenging activities in ethanol bark extracts of* X. granatum *(XGEB). Administration of XGEB at 100 and 200 mg/kg BW doses to STZ-induced diabetic mice resulted in significant decrease (P < 0.05) in blood glucose, triglyceride (TG), total cholesterol (TC), serum glutamate oxaloacetate transaminase (SGOT), serum glutamate pyruvate transminase (SGPT), and urea levels in the serum of the extract administered groups as compared to diabetic control group. The levels of SOD, CAT, GPx, GR, and GST in liver along with LPx, SOD, GST, and GR activities in brain tissues were found to be ameliorated in XGEB treated diabetic mice. Histopathological alternations of liver tissues were also found to be restored in XGEB treated diabetic groups. The HPLC fingerprint analysis of XGEB revealed the presence of simple polyphenols, isoflavone, and flavonol-like compounds. The DSC and UV-VIS analysis also confirmed the presence of phenolic compounds in XGEB. The GC-MS analysis of XGEB showed the presence of a number of bioactive compounds. These results demonstrated the beneficial effect of XGEB in controlling hyperglycaemia and ameliorating oxidative stress associated complications associated with diabetes.

## 1. Introduction

Diabetes mellitus is a multifactorial metabolic syndrome characterized by defect in the secretion of insulin associated with deregulation in carbohydrate, protein, and lipid metabolism. It is one of the most prevalent diseases affecting all ages of people across the globe. It affected an estimated 415 million people in 2015 and is expected to increase up to 642 million by 2040 [1]. Increasing evidence in both experimental and clinical studies suggests that oxidative stress plays an important role in the pathogenesis of diabetes mellitus. In diabetic condition, free radicals are formed disproportionately mainly due to glucose oxidation and nonenzymatic glycation of proteins leading to depletion of endogenous antioxidant components resulting in increased oxidative stress and development of insulin resistance [2]. Although several hypoglycaemic drugs are available for treatment of diabetes, they have side effects and usually fail to alleviate oxidative stress and its associated complications. Therefore, management of diabetes without side effects remains a challenge. On the other hand medicinal plants with antidiabetic and antioxidant properties can serve as ideal phytotherapy for treatment of diabetes and oxidative stress associated complications. In this context, mangrove plants growing in the stressful environment at the interface of sea and land can be an important source of drug for treatment of diabetes due to its antidiabetic as well as antioxidant properties because of possession of rich secondary metabolites.

*Xylocarpus granatum *J. Koenig (Meliaceae) is an important medicinal mangrove plant and well distributed in a number of countries of south-east Asia, Australia, and east Africa [3]. Different parts of this plant have been used traditionally as astringent and febrifuge along with treatment of fever, malaria, thrush, cholera, dysentery, and diarrhoea in many countries including India [4]. Earlier studies have reported the free radical scavenging properties of leaves and barks extracts of* X. granatum* [5]. The epicarp of fruit extracts of this plant has also reported for antidiabetic and antidyslipidaemic properties [6]. Secondary metabolites of different classes have been reported in* X. granatum* such as limonoids (gedunin, xyloccensins, xylograntins, xylocarpins, and xylomexicanins), catechin, epicatechin, 6-dehydroxyxylocarpin D, kaempferol 3-*O*-*β*-D-glucoside, ergosterol peroxide, *β*-sitosterol, daucosterol, 4-hydroxybenzoic acid, ethyl 3,4-dihydroxybenzoate, carapolide-A,B, alkaloids, harzianone, trichoacorenol, and trichodimerol [7, 8].

The preliminary study by the authors revealed the higher* in vitro* antidiabetic and antioxidant activities of ethanol bark extract of* X. granatum* as compared to leaves extracts [9]. However, no reports are available on the protective effect of the ethanol bark extract of* X. granatum *(XGEB) on hyperglycaemia mediated oxidative stress in STZ-induced diabetic mice. Therefore, the present study is aimed to undertake a detailed investigation on* in vivo* evaluation of ethanol bark extracts of* X. granatum* (XGEB) on hyperglycaemia and its oxidative stress associated complications in STZ-induced insulin-dependent diabetic mice model. Further, the gas chromatography mass spectroscopy (GC-MS) analysis of ethanol bark extract of* X. granatum *was conducted to identify the major bioactive compounds.

## 2. Materials and Methods

### 2.1. Chemicals

p-Nitrophenyl-*α*-D-glucopyranoside (pNPG), Folin-Ciocalteu's phenol reagent (FCP), catechol, catechin, potassium persulfate, ferrous sulphate, sodium potassium tartrate, 3,5-dinitrosalicylic acid (DNS), sodium hydroxide, 2,2'-azino-bis(3-ethylbenzothiazoline-6-sulphonic acid) (ABTS), Butylated hydroxy toluene (BHT) and *α*-amylase, streptozotocin (STZ), and ferrozine were purchased from SRL India, Ltd. Acarbose was purchased from Sigma Aldrich India. All the chemicals and reagents used in the study were of analytical grade.

### 2.2. Collection of Plant Material

Leaves and barks of* X. granatum *(family-Meliaceae) were collected from the mangrove forest of Mahanadi delta area of Odisha coast (India). The specimen was authenticated by Prasanna Kumar Nayak, Herbarium keeper, Integrated Coastal Zone Management Project (ICZMP), Forest Department, Govt. of Odisha, India. The specimens were identified at Department of Natural Products, Institute of Minerals and Materials Technology, Bhubaneswar (RRL-B), Odisha, India, and voucher specimen (VS No. RRL-B 12567) was deposited.

### 2.3. Extraction of X. granatum

Successive Soxhlet extraction method was followed to prepare crude extracts from leaves and barks of* X. granatum*. The leaves and barks plant materials were dried under shade and pulverized. The pulverized plant materials (20g) were then extracted successively with 200 ml of 90% ethanol, methanol, and water in Soxhlet apparatus [10]. After extraction, the extracts were concentrated under reduced pressure in rotary evaporator (IKA- RV10).

### 2.4. In Vitro Antioxidant and Antidiabetic Activities

The* in vitro* antioxidant activities of the ethanol, methanol, and aqueous leaves and barks extracts were evaluated by ABTS scavenging [11] and metal chelating assay [12] and* in vitro* antidiabetic activities by *α*-amylase inhibition assay [13]. The antioxidant assay was carried out using standard antioxidant compound Butylated hydroxy toluene (BHT). Similarly, the* in vitroα*-amylase inhibition assay was carried out using standard antidiabetic compound Acarbose.

### 2.5. Experimental Animals

A total of 30 healthy, adult male Balb/c mice of 2 months of age and average body weight of 30 g were maintained under controlled conditions of temperature (23±2°C) and humidity and a 12 h light-dark cycle and were used for the experiment. They were housed in sanitised polypropylene cages and had free access to standard mice pellet diet and water* ad libitum*. All the experimental procedures were performed in IMGENEX India Pvt. Ltd. (No. 526/CPCSEA; 21-01-2002) in accordance with the Committee for the Purpose of Control and Supervision of Experiments on Animals (CPCSEA).

### 2.6. Induction of Diabetes

STZ was dissolved in 0.05 M citrate buffer (pH 4.5) and injected intraperitoneally (*i.p.*) to overnight fasted mice at a single dose of 125 mg/kg body weight. The animals were allowed to drink 5% glucose solution to overcome the drug induced hypoglycemia. After 72 h of STZ administration, blood samples were collected from tail and glucose levels were estimated by glucostrips (One Touch Glucometer, Life Scan, Europe). Mice having fasting blood glucose levels above 200 mg/dl were considered diabetic and subsequently used in the present study.

### 2.7. Acute Toxicity Study

The toxicity of the extract was assessed as per the previously described method. [14]. Healthy Balb/c mice were randomly assigned into three groups and were given the XGEB extracts at the doses of 100, 300, and 1000 mg/kg body weight orally daily for 4 days by dissolving in 0.05 M citrate buffer (pH 4.5) as vehicle. The animals were then observed for 96 h. Behaviour signs were recorded.

### 2.8. Experimental Design

After acclimatization mice were divided into six groups of five animals each.

NC group: normal control mice supplemented with vehicle (0.05 M citrate buffer, pH 4.5);

NCT: normal mice treated with ethanol bark extracts of* X. granatum* (200 mg/kg);

DMC: diabetic control mice supplemented with vehicle;

DMD: diabetic mice treated with glibenclamide drug (3 mg/kg);

DML: diabetic mice treated with ethanol bark extracts of* X. granatum* (100 mg/kg);

DMH: diabetic mice treated with ethanol bark extracts of* X. granatum* (200 mg/kg).

The glibenclamide and* X. granatum* ethanol bark extracts were dissolved in 0.05 M citrate buffer (pH 4.5) and administered orally daily for 30 days. On 0, 7^th^, 14^th^, 21^st^, and 30^th^ day, the body weights of mice were recorded and blood samples were collected from each animal by puncturing the tail veins. At the end of the experiment, blood was collected from the mice by retroorbital bleeding. Liver and brain tissues (cerebral cortex) were excised immediately after sacrificing mice and stored at -20°C till further use.

### 2.9. Biochemical Analysis

Different parameters like total cholesterol (TC), triglycerides (TG), urea, serum glutamate oxaloacetate transminase (SGOT), and serum glutamate pyruvate transminase (SGPT) levels were determined using the commercial kits (Tulip Diagnostics, India). The liver and brain tissues were assayed for activities of different parameters like lipid peroxidation (LPx), reduced glutathione content (GSH), superoxide dismutase (SOD), catalase (CAT), glutathione peroxidase (GPx), glutathione reductase (GR), and glutathione-s-transferase (GST) employing established experimental methods [15–20].

### 2.10. Histopathology of Liver

The liver tissues from each animal were collected and small pieces of each tissue were fixed in sublimate formol and processed by the paraffin technique. Thin sections (7 *μ*m) were cut and stained with hematoxylin-eosin as per the routine staining method. The tissue samples were then examined and photographed under a light microscope [21]. Different changes in the liver tissue such as degeneration of hepatocytes, fatty changes of hepatocytes, inflammatory cell infiltrations, and sinusoidal dilation were evaluated. Scoring of the histopathological changes was done as either present (+) or absent (-).

### 2.11. Phytochemical Analysis

A quantitative phytochemical test for determination of flavonoid and tannin content of the leaf and bark extracts of* X. granatum* were carried out using standard procedures [22].

### 2.12. UV-VIS Spectral Analysis

The UV–VIS spectrophotometer UV–117 (Systronics™) was used to measure UV–VIS absorbance spectra of different extracts of* X. granatum* [23]. The absorbance measurements were measured in 200 –700 nm range with a 1 nm step and the characteristic peaks were detected.

### 2.13. HPLC Analysis

For phytochemical fingerprinting, the ethanol bark extract of* X. granatum* was analyzed by high performance liquid chromatography (HPLC) (Analytical Technologies, Baroda, India) technique following the method described by Kumar et al. (2008) [24]. Simultaneous rapid separation of polyphenols was performed by using RP-C18 Lichrocart 250-4, 5 *μ*m (250 x 4.6 mm) as stationary phase and a linear gradient elution is carried out by mobile phase (water and acetonitrile acidified with 0.02% trifluoroacetic acid) starting with 80% and ending with 20% water in total 22 min run time. The flow rate of mobile phase was kept at 1 ml min^−1^. The detection was carried out at 280 nm using an UV detector.

### 2.14. Differential Scanning Calorimetric Analysis

The differential scanning calorimetry (DSC) curves were obtained on a TA Instruments Calorimeter, model DSC 4000 Perkin Elmer, Singapore. The analysis was performed using aluminium crucibles with about 2 ± 0.1mg of samples under nitrogen atmosphere, at a flow rate of 20 ml/min. DSC thermogram was recorded constantly and continuously by increasing the temperature from 20 to 300°C at a heating rate of 20°C/min. Indium (m.p. 156.6°C) was used as standard for equipment calibration [25].

### 2.15. GC-MS Analysis

The ethanolic bark extract of* X. granatum* was analyzed using GC-MS [26]. The analysis was conducted with an Agilent Technologies 7890B GC ION TRAP MS (Agilent Technologies, Santa Clara, California, USA). ADB-5SILMS capillary column was used (30 m x 0.25 mm internal diameter, 0.25 *μ*m film thickness). The ultrapure helium was used as a carrier gas at a flow rate of 0.7 mL/min and a linear velocity of 37 cm/s. The injector temperature was set at 250°C. The initial oven temperature was 60°C, which was ramped up to 280°C at a rate of 10°C/min with a hold time of 3 minutes. The MS operating conditions were electron ionization mode at 70 eV and scan range 50-700 amu. Compounds were identified by comparing the retention times and mass fragmentation with the National Institute of Standards and Technology (NIST) library.

### 2.16. Statistical Analysis

The data were analyzed using the SPSS for Windows, version 20, IBM Corporation. Statistical analysis was done by two-way ANOVA for blood glucose level, whereas other biochemical parameters were analysed by one-way ANOVA, followed by LSD. Experimental data were expressed as mean ± standard deviation (SD). A level of P < 0.05 was accepted as statistically significant.

## 3. Results

### 3.1. In Vitro Antioxidant and Antidiabetic Activities

Table 1 demonstrates the* in vitro* antioxidant and antidiabetic activities of leaf and bark extracts of* X. granatum* in terms ABTS radical scavenging and *α*-amylase inhibitory activities, respectively. The ethanol bark extracts of* X. granatum* (XGEB) exhibited higher antioxidant and antidiabetic activities (expressed in terms of IC_50_ values) as compared to other solvent extracts of leaf and bark. The ethanol bark extract showed highest ABTS scavenging activity with IC_50_ value of 41.50 *μ*g/ml. Under the similar condition the standard antioxidant compound Butylated hydroxyl toluene (BHT) showed antioxidant activity with 76.34 *μ*g/ml. The highest *α*-amylase inhibitory activity was also observed in ethanol bark extract with IC_50_ value of 0.36 mg/ml. Under the similar condition, the standard drug Acarbose could inhibit the *α*-amylase enzyme with IC_50_ value of 0.15 mg/ml (Table 1). However, none of the extracts showed any metal chelating activity at 50, 100, or 150 *μ*g/ml concentration.

### 3.2. Acute Toxicity Study

Oral administration of XGEB extracts at a dose of 1000 mg/kg body weight/day did not produce any signs of toxicity and no animals died up to 4 days. It showed that XGEB was nontoxic in mice up to an oral dose of 1000 mg/kg body weight. However, further investigation of antidiabetic and antioxidant activities was carried out using 100 and 200 mg/kg body weight dose levels where a significant lowering of blood glucose level in comparison to diabetic control was observed.

### 3.3. Changes in Body Weight

Changes in initial and final body weight in control and experimental groups were shown in Table 2. The body weight of DMC group was decreased significantly (p<0.05) as compared to NC group. The body weight of DMC at the end of 30^th^ day declined by 19.64% as compared to day 1. The body weight of DMD and DMH increased significantly (p<0.05) as compared to DMC group whereas the body weight of DML group decreased (9.06%) during 30-day experiment period. The increase in body weight for NC, NCT, DMD, and DMH groups at the end of experiment (30^th^ day) was 17.82%, 25%, 13.67%, and 4.59%, respectively, as compared to day 1.

### 3.4. Blood Glucose Level

The blood glucose level of DMC mice was increased significantly (p<0.05) after STZ-induction as compared with normal control mice. The DMC group displayed 311.4 ± 37.87 mg/dl glucose on day 1 which was increased to 393.3 ± 32.07 mg/dl on day 30 accounting an increase in 27.19% (Table 3). However, oral administration of glibenclamide and XGEB to diabetic mice significantly reduced (P <0.05) the blood glucose level in as compared with diabetic control mice. As shown in Table 3, after 30 days of treatment, the STZ-induced hyperglycaemia was significantly ameliorated by XGEB extracts which was related to dose and duration of treatment. The XGEB extract at 100 mg/kg and 200 mg/kg body weight reduced the blood glucose level by 19.58% in DML and 31.21% in DMH groups, respectively. No significant deviation was observed in normal mice treated with XGEB.

### 3.5. Biochemical Parameters of Blood

The TG and TC levels were increased significantly (p< 0.05) in DMC group mice as compared to NC group mice (Table 4). On the other hand, the administration of glibenclamide or XGEB (bothat 100 mg/kg and 200 mg/kg body weight) showed significant reduction (p< 0.05) in TG and TC levels in DMD, DML, and DMH groups as compared to DMC group. The TG levels were decreased by 32%, 16%, and 28%; while the TC levels were decreased by 36%, 35%, and 39% in DMD, DML, and DMH groups compared to DMC group.

The SGOT, SGPT, and urea levels were increased significantly (p< 0.05) in DMC group as compared to NC group (Table 4). Upon oral administration of glibenclamide and XGEB a significant reduction (p< 0.05) in SGOT, SGPT, and urea levels was observed in DMD, DML, and DMH groups in comparison to DMC group. The SGOT levels were decreased by 35%, 25%, and 39%; the SGPT levels were decreased by 28%, 16%, and 35% while the urea levels were decreased by 34%, 14%, and 32% in DMD, DML, and DMH groups as compared to diabetic control mice.

### 3.6. LPx and GSH Content in Liver and Brain

Significantly higher (p<0.05) liver LPx level was found in diabetic control mice as compared to normal mice (Table 5). However, the treatment with XGEB or glibenclamide did not show any change in liver LPx levels. The LPx level in brain tissue remained unchanged in DMC mice as compared to NC mice. However, treatment with XGEB and glibenclamide resulted in marked decrease (p<0.05) in the brain LPx levels compared to DMC group. The LPx levels in brain tissues were decreased by 62%, 23%, 49% DMD, DML, and DMH groups as compared with DMC mice.

The activities of nonenzymatic antioxidants such as NP-SH and P-SH in liver and brain tissues of the normal and diabetic mice are summarized in Table 5. The P-SH content of liver was significantly decreased (p< 0.05) in diabetic control mice as compared to normal mice. The NP-SH content of brain was significantly increased in XGEB treated (200 mg/kg bw) groups as compared to diabetic control mice. However, NP-SH content in liver along with P-SH and NP-SH contents in brain remained unchanged in diabetic control mice as compared to control mice. Administration of neither XGEB nor glibenclamide could ameliorate the NP-SH and P-SH levels in liver and brain tissues of diabetic mice except for NP-SH level in brain tissue of DMH mice where a significant (p<0.05) increase is observed as compared to diabetic control mice.

### 3.7. In Vivo Antioxidant Levels

The activities of enzymatic antioxidants such as SOD, CAT, GPx, GST, and GR in liver and brain tissues of the normal and diabetic mice are summarized in Table 6. The SOD (liver and brain), GR (liver and brain), and GST (liver and brain) levels were significantly decreased (P< 0.05) in diabetic control mice as compared to normal control mice. But the decrease in CAT level in liver tissue was nonsignificant (P>0.05) as compared to normal control. Upon administration of glibenclamide and XGEB to diabetic mice, the activities of SOD and GR levels in liver tissues increased significantly (P<0.05) in treated mice as compared to diabetic control mice. Similarly, significant (P<0.05) increase in CAT level was observed in liver tissue of XGEB treated (200 mg/kg bw) mice as compared to diabetic control mice. But the increase in CAT level was nonsignificant in liver tissues of XGEB treated (100 mg/kg bw) and glibenclamide treated mice as compared to diabetic control mice. The GR and GST level in brain tissues were increased significantly (P<0.05) in both glibenclamide treated and XGEB (200 mg/kg bw) treated mice as compared to diabetic control mice whereas the increase was found to be nonsignificant (P>0.05) in XGEB (100 mg/kg bw) in treated mice. However, nonsignificant (P>0.05) increase in SOD level in brain tissues of both XGEB and glibenclamide groups mice was recorded as compared to diabetic control mice group.

### 3.8. Histopathological Analysis of Liver

**Figure 1:**
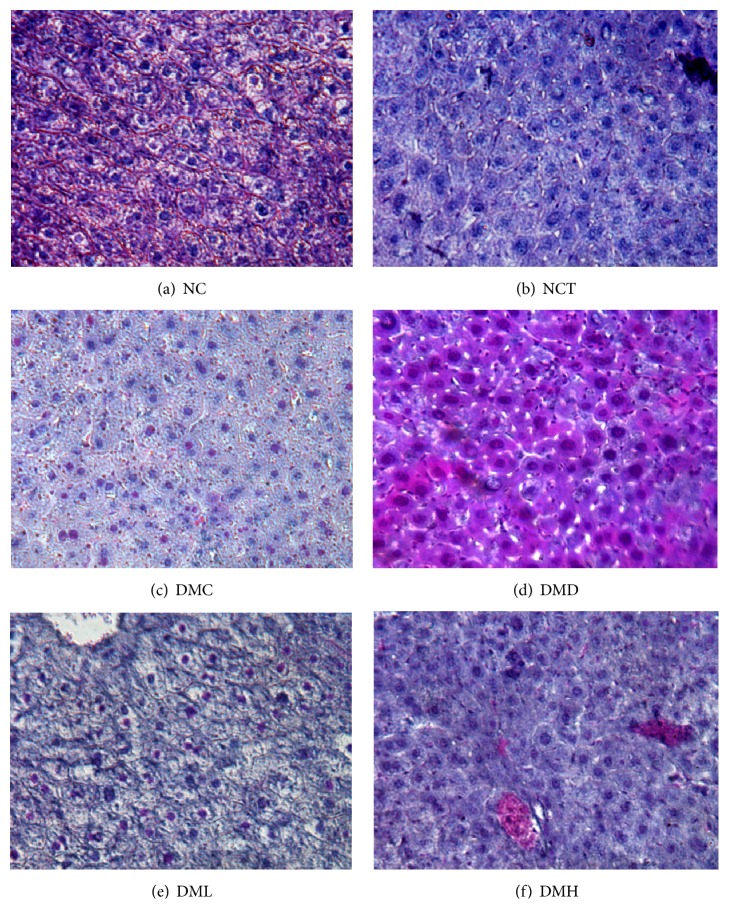
Representative photomicrograph showing histopathology of liver. (a) normal control mice (NC); (b) normal control toxicological dose (NCT); (c) diabetic control mice (DMC); (d) diabetic + glibenclamide (DMD); (e) diabetic +* X. granatum* low dose (DML); (f) diabetic +* X. granatum* high dose (DMH). Magnification x 40.

The histopathological analysis of the liver in NC and NCT groups showed normal cell morphology with hexagonal lobular architecture. However, the liver sections in diabetic control mice showed progressive disruption of structural architecture characterized by an apparent decrease in number of intracytoplasmic organelles, inflammatory damage, sinusoidal dilation, and fatty changes. The sections of glibenclamide treated diabetic mice showed restoration of architecture of hepatocytes. However, moderate sinusoidal dilation and inflammatory damage were observed. On the other hand, liver sections from XGEB treated diabetic mice showed reduced histopathological damages as compared to diabetic control group (Figure 1). Inflammatory damages were not observed. The fatty changes of liver and sinusoidal dilation were not observed. The semiquantitative histological scoring of liver damage is presented in Table 7.

### 3.9. Phytochemical Analysis

Quantitative phytochemical screening showed that the aqueous leaf extract possessed highest amount of total flavonoid content (10 mg QE/g DW) amongst the different extracts of* X. granatum*. Similarly, amongst the different extracts, the ethanol bark extract, was found to possess highest total tannin content, i.e., 9.76 mg GAE/g DW (Table 8).

**Figure 2:**
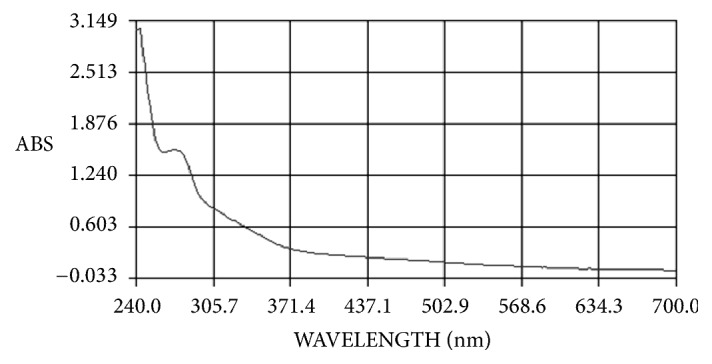
UV-VIS Spectra of ethanol bark extract of* X. granatum*.

The ethanol bark extracts of* X. granatum* demonstrated highest antidiabetic and antioxidant activities amongst all the extracts studied, hence chosen for further phytochemical analysis to obtain some information on the active components present in extract. The ethanol bark extract was subjected to UV-VIS, HPLC, DSC, and GC-MS analysis. The UV-Visible absorbance profile of the ethanol bark extracts of* X. granatum* was studied for detection of phenolic compounds at a wave length range of 200 to 700 nm (Figure 2). The spectrum showed an absorbance maximum at 274 nm for ethanol bark extracts with the absorption values of 1.563 indicating the presence of phenolic acid derivatives.

**Figure 3:**
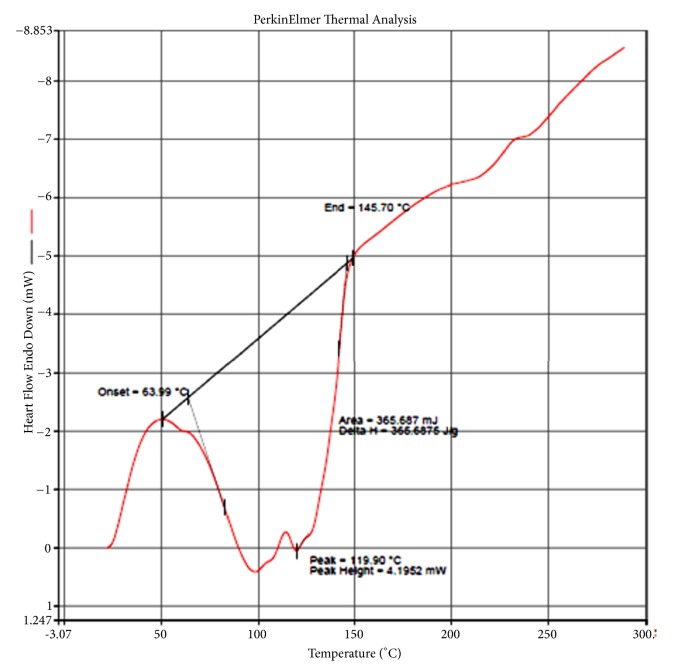
DSC curve for ethanol bark extract of* X. granatum*.

Further, the purity and thermal behaviour of the sample was studied by differential scanning calorimetric method. The DSC thermogram of XGEB showed a broad peak at 119.9°C with onset and end at 63.99°C and 145.7°C (Figure 3). The heat of fusion for the XGEB was found to be 366 J/g. An additional peak at 100°C along with the peak at 119.9°C was also observed due to the loss of hydroxyl functional group as water which may be due to the presence of phenolic compounds in the ethanol bark extract.

**Figure 4:**
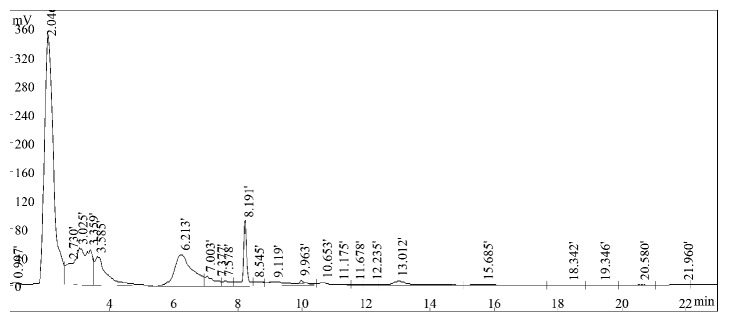
HPLC chromatogram of ethanol bark extracts of* X. granatum*.

For phytochemical fingerprint, the ethanol extracted bark sample of* X. granatum *was analyzed by high performance liquid chromatography (HPLC) which gave five major peaks at Rt 3.02, 3.58, 6.21, 8.19, and 13.01 min indicating the presence of simple polyphenolic compounds, isoflavone and flavonol (Figure 4).

**Figure 5:**
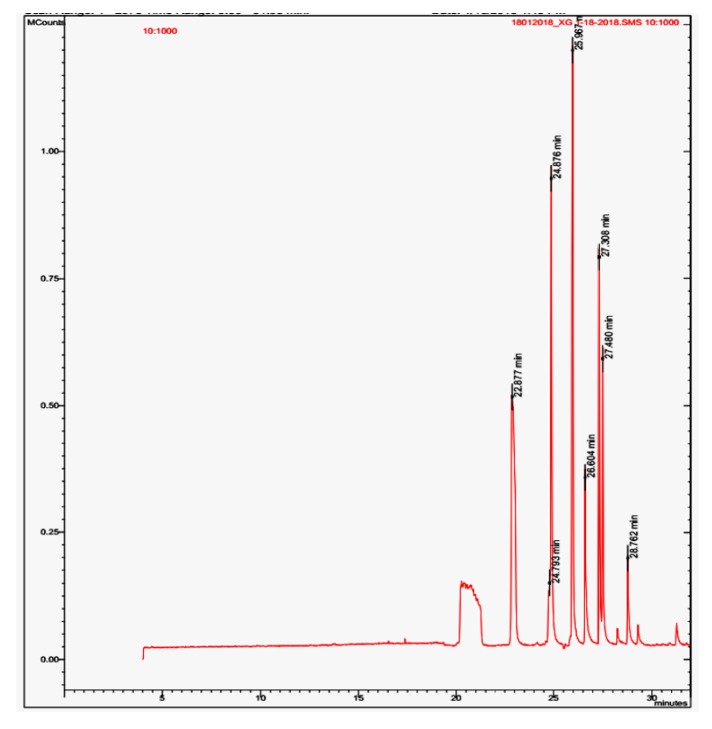
GC-MS analysis of ethanol bark extract of* X. granatum*.

The GC-MS analysis of the ethanol bark extract of* X. granatum* indicated the presence of 13 peaks out of which 8 peaks were characterized on the basis of their retention time and five peaks such as 2, 5, 6, 7, and 12 were not characterized (Figure 5). The compounds identified were phenol, 2,4-bis (1,1-dimethylethyl) [peak 1], tetracosamethyl cyclododeca siloxane [peak 3], bis(p-(phenylethynyl)phenyl) butadiyne [peak 4], 6,6'-diacetyl-7,7'-dihydroxy-2,2',4,4',5 [peak 8], 6,6'-diacetyl-7,7'-dihydroxy-2,2',4,4',5 [peak 9], bis(heptamethyl cyclotetrasiloxy) hexameth [peak 10], 3-phorbinepropanoic acid, 9-acetyl-14-et [peak 11], and phenol, 4,4'-methylene bis [2,6-bis (1,1-di) [peak 13].

## 4. Discussion

The present study has made a novel attempt to evaluate the antidiabetic and antioxidant properties ethanol bark extract of* X. granatum, *a mangrove plant by both* in vitro* and* in vivo* studies. Results from the present* in vitro* antidiabetic investigation have demonstrated that the ethanol, methanol and aqueous extracts of leaves and bark of* X. granatum* possess *α*-amylase inhibition activity with the highest potency noted in the ethanol bark extracts. Previously, the leaf and bark extracts of this plant have also been reported for their glucose uptake capacity and *α*-glucosidase inhibition property [6]. Inhibition of *α*-amylase and *α*-glucosidase is an effective strategy for prevention of diabetes as they play important roles in controlling postprandial blood glucose level by delaying carbohydrate digestion and consequently blunting the postprandial plasma glucose rise [27]. Inhibition of *α*-amylase and *α*-glucosidase enzymes have also been reported in several other mangrove plants like* Barringtonia racemosa*,* Rhizophora mucronata*,* Ceriops tagal*,* Sonneratia caseolaris* suggesting the presence of antidiabetic bioactive principles in mangrove plants which have therapeutic implications [27–30].

Postprandial hyperglycaemia is a common pathogenesis in diabetes incurred due to insulin resistance and *β*-pancreatic destruction [31]. In the present study, treatment with XGEB, at both 100 and 200 mg/kg, resulted in a significant (P< 0.05), consistent, and dose dependent decrease in blood glucose level throughout the experimental period indicating its potent antidiabetic activity. The result could be linked to the potent *α*-amylase and *α*-glucosidase inhibitory activity of XGEB that could cause decrease in the digestion of carbohydrates. Decrease in body weight is often found to be associated with diabetic conditions because of increase in muscle wastage, decrease in tissue proteins, and breakdown fat [32]. In the present study, XGEB at the dose level of 200 mg/kg showed an improvement in body weight gain as compared to diabetic control group suggesting the restorative effect of XGEB extract which may be due to the reversal of gluconeogenesis and glycogenolysis.

Uninhibited actions of lipolytic hormones on fat cells due to impairment of insulin secretions result in hypertriglyceridemia and hypercholesteromia in diabetes that further increase the risk of cardiovascular diseases [33]. In the present study, administration of XGEB at 100 and 200 mg/kg to the diabetic mice significantly (P< 0.05) improved the TG and TC levels towards normalcy which may be due to the decreased cholesterogenesis and enhanced glucose utilization. These results imply that XGEB administration could effectively improve the metabolism of carbohydrates, lipids in diabetic patients. The urea and creatinine levels in the blood are considered as one of the noticeable indices for renal function under diabetic condition [34]. Significant (P< 0.05) decrease in the blood urea level in diabetic mice treated with XGEB (at 100 and 200 mg/kg) indicated that XGEB extract prevents the progression of renal damage in STZ-induced insulin-dependent diabetic mice.

The liver is the vital organ for metabolism and detoxification of xenobiotics. During diabetes, the liver cells are necrotized and released the liver enzymes like SGOT, SGPT, and alkaline phosphatase (ALP) into blood stream leading to increase in their concentration [35]. In the current study, reduction in SGOT and SGPT levels in blood of XGEB treated diabetic groups signified the hepatoprotective of* X. granatum* ethanol extracts. Therefore, restoration of these enzyme biomarker enzymes towards normal level indicate decreased diabetic complications in XGEB treated diabetic mice. Histopathological examination of liver also showed a similar effect. As per the histopathological results, XGEB extract (at both 100 and 200 mg/kg dose) could decrease sinusoidal dilation and inflammatory cell infiltration along with amelioration of degeneration and fatty changes of hepatocytes as compared to the diabetic control group. These results revealed that active components of XGEB extract could diminish oxidative stress, which was induced by STZ in the diabetic mice. Further, supplementation of XGEB to normal mice did not show much alteration in SGOT, SGPT enzyme activities indicating that XGEB administration was safe and possessed no significant toxicity. The ability to maintain the renal and hepatic factors close to the normal conditions supports the ability of the extract to protect them from nephropathy and hepatopathy.

During diabetes, persistent hyperglycaemia impairs prooxidant and antioxidant balance that reduces antioxidant level and increases production of ROS. Oxidative stress due to free radical generation and reduction of endogenous antioxidant is considered as one of the underlying factors in the development of diabetes complications. Hyperglycaemia induced oxidative stress leads to the activation of stress pathways which ultimately lead to tissue damage increasing lipid peroxidation that further impairs glucose metabolism in biological systems [36]. In agreement with the above study, a significant increase in the LPx level was observed in the liver of diabetic mice in the current study. However, neither glibenclamide nor the XGEB extract was able to bring back the elevated LPx level to control. At the same time, it is noticed that both the drug and the plant extract were able to maintain the LPx level without further increase during the treatment period. Though brain is rich in fatty acids; the level of Lpx remained unaltered in diabetic mice but reduced significantly in drug and extract treated groups. Therefore, it will not be out of context to mention that both the drug and the plant extract might be exerting their tissue specific differential effects by modulating the activities of cellular antioxidant enzymes or by scavenging ROS generated due to diabetes after treatment or by maintaining a stable glucose level. In fact we have noticed marked alterations in the activities of antioxidant enzymes in the liver and brain of mice in response to drug and XGEB extract treatment.

Antioxidant enzymes act in a cascade. SOD dismutates O_2_^.-^ to H_2_O_2_. H_2_O_2_ which is not very favorable to the cell is neutralized by CAT and GPx into O_2_ and H_2_O. The Km for H_2_O_2_ is > 10 mM in mammalian cells. Therefore, at low intracellular concentrations GPx is the pivotal enzyme for degradation of H_2_O_2_ [37]. In the present study, a significant decrease in the activity of SOD along with an augmentation in GPx activity is noticed in both liver and brain tissue of diabetic control mice. A decreased SOD activity in the tissues will lead to increase in O_2_^.-^ content of the cell which is capable of generating ^.^OH in presence of transition metal ions such as iron or peroxynitrite in presence of NO. The highly reactive ^.^OH radical has the ability to attack any biomolecule within 1 atomic radius and thus oxidizing lipids and proteins and inducing strand breaks in DNA leading to cellular dysfunction. To corroborate the fact, enhanced LPx level was noticed in the liver in diabetic mice.

In case of liver, administration of XGEB at 200 mg/kg to diabetic mice showed a marked increase in SOD and CAT activity and brought back the level of GPx and GR to normal level, thus showing an enhancement of antioxidant defense in response to diabetic induction. This fact is further corroborated by decline in GST activity which is known to be induced by toxic xenobiotics and oxidative stress [38]. Glibenclamide and the lower dose of XGEB extract showed less impact on antioxidant defense. The decrease in NP-SH in extract treated diabetic mice may be due to direct scavenging of ROS by the antioxidants present in the extract thus circumventing the production of NP-SH more precisely GSH which is further corroborated by the decline in principal GSH metabolizing enzymes GPx and GST. Similarly, the lowered P-SH level indicates an adaptive response to protect proteins in response to diabetes induced ROS generation. In case of brain, the altered activities of SOD, GPx, and GST enzymes in diabetic mice brought back to the normal control level after administration of* X. granatum* at 200 mg/kg except GR which was elevated. This might be an adaptive response of brain to protect itself from ROS mediated cell damage [39].

As discussed earlier, this property of XGEB highlights that the activity could be due to either some potential antioxidant compounds or other biomolecules which could alone or synergistically act with the antioxidants present in the extract. The antioxidant action of XGEB may be attributed to the presence of antioxidant components or due to other biomolecules which could alone or synergistically act with the antioxidants present in the extract. In fact results from the present study on free radical scavenging activity using ABTS scavenging assay demonstrated that amongst all the extracts of leaves and barks of* X. granatum*, the ethanol bark extracts exhibited highest ABTS scavenging potential. This action could be beneficial for eliminating ROS and in turn attenuate the complications of diabetes.

The quantitative phytochemical assays also showed the presence of flavonoids and tannins in leaf and bark extracts of* X. granatum*. The HPLC fingerprinting is one of the simplest ways for chemical characterization of bioactive compounds from plants and their extracted fractions. According to fingerprint developed by Kumar et al. (2008) [24] simple polyphenols like gallic acid elutes first at Rt 3.63 min. After gallic acid, catechin (Rt 4.57) and epicatechin (Rt 5.24) group compounds are eluted in the mobile phase. Rutin belongs to isoflavone group eluted after catechins at 6.94 min, compounds of flavonol groups eluted in between Rt 9 to 16 min. Phytochemicals fingerprint of ethanol bark extract of* X. granatum* plant was compared with above-mentioned fingerprint under similar chromatographic conditions. Ethanolic extract of* X. granatum* bark shows well developed chromatographic peak at Rt 3.02, 3.58 min which represent the presence of simple polyphenolic compounds. This is followed by peaks at Rt 6.21 and Rt 8.19 min which represents isoflavone. Chromatographic peak at R t 13.01 min was also observed which represents the presence of flavonol in the extract. The present study indicated the presence of simple polyphenols, isoflavone, and flavonol in the ethanol bark extract of* X. granatum.* The DSC thermogram of* X. granatum *also indicated the presence of phenolic compounds in the ethanol bark extract [25]. The UV-VIS spectral analysis also confirmed the presence of phenolic derivatives in the ethanol bark extract [40]. The GC-MS analysis of the ethanol bark extract of* X. granatum* indicated the presence of different bioactive compounds such as phenol, 2,4-bis (1,1-dimethylethyl); bis(p-(phenylethynyl)phenyl) butadiyne; 6,6'-diacetyl-7,7'-dihydroxy-2,2',4,4',5; 6,6'-diacetyl-7,7'-dihydroxy-2,2',4,4',5; bis(heptamethyl-cyclotetrasiloxy) hexameth; 3-phorbinepropanoic acid, 9-acetyl-14-et; and phenol, 4,4'-methylene bis [2,6-bis (1,1-di) out of which 2,4-bis (1,1-dimethylethyl) phenol, tetracosamethylcyclododecasiloxane have been reported for their antioxidant properties in some previous studies [41, 42]. The antidiabetic potential of plant species has been tested in nicotinamide induced Type-2 diabetic rats [43] and the present studies also prove that plants are the rich source of natural antidiabetic compounds.

## 5. Conclusion

The results of the present study clearly indicated that the ethanol bark extracts of* X. granatum* possess antioxidant and antidiabetic potentials. The XGEB exerts its antioxidant effect by scavenging the free radicals and thereby regulates the antioxidant status in STZ-induced diabetic mice. The antidiabetic potentials of XGEB were also comparable with the antidiabetic drug glibenclamide. The presence of different phenolic derivatives in XGEB may act as potential candidates in counteracting the oxidative damage and inhibiting the progression of diabetes and its associated complications. Therefore, the* X. granatum* bark supplementation may be helpful in management of diabetes complications. However, in-depth study is warranted to isolate the bioactive components and to elucidate their exact mechanism of action by which this plant regulates glucose homeostasis.

## Figures and Tables

**Table 1:** ABTS radical scavenging (expressed as IC_50_ value in *µ*g/mL) and *α*-amylase inhibitory activities (expressed as IC_50_ value in mg/mL) of *X. granatum* extracts. EL = Ethanol leaf extracts; ML = Methanol leaf extracts; AL = Aqueous leaf extracts; EB = Ethanol Bark extracts; MB = Methanol Bark extracts; AB = Aqueous Bark extracts of *X. granatum*. The values are expressed as mean ± SD (n=3).

Sample	ABTS	*α*-Amylase
EL	42.02 ± 0.41	1.04 ± 0.05
ML	127.54 ± 1.34	0.95 ± 0.12
AL	43.30 ± 1.16	2.22 ± 0.03
EB	41.50 ± 0.9	0.36 ± 0.01
MB	43.29 ± 0.84	0.42 ± 0.03
AB	59.89 ± 0.89	0.99 ± 0.13
Standard	76.34 ± 0.66	0.15 ± 0.02

**Table 2:** Effect of *X. granatum* ethanol bark extracts on body weight. Data are expressed as mean ± SD (n= 5). Data in parentheses indicate percent gain (+) or loss (-) in weight. NC, Normal Control; NCT, Normal Control Toxicological (high) dose; DMC, Diabetic Control; DMD, Diabetic Drug (Glibenclamide); DML, Diabetic *Xylocarpus granatum* Low dose (100 mg/kg).; DMH, Diabetic *Xylocarpus granatum* High dose (200 mg/kg). ^a^p<0.05 with respect to initial body weight in the same group. ^b^p<0.05 with respect to final body weight of NC, ^c^p<0.05 with respect to final body weight of DMC.

Groups	Mean body weight (g)	
	*Initial (1* ^*st*^ *Day)*	*Final (30* ^*th*^ *Day)*
NC	37.2 ± 2.38	43.83 ± 1.92^a^
		(+ 17.82%)
NCT	33.2 ± 2.94	41.5 ± 4.38^a,c^
		(+ 25%)
DMC	28 ± 1.87	22.5 ± 1.11^a,b^
		(-19.64%)
DMD	32.9 ± 2.01	37.4 ± 1.71^a,b,c^
		(+ 13.67%)
DML	34.2 ± 6.30	31.1 ± 6.93^a,b,c^
		(-9.06%)
DMH	33.94 ± 3.26	35.5 ± 2.69^a,b,c^
		(+ 4.59%)

**Table 3:** Effect of *X. granatum* ethanol bark extracts on blood glucose level. Data are expressed as mean ± S.D. (n=5). Data in parentheses indicate % increase. NC, Control mice; NCT, Normal Control mice +Toxicological (high) dose *Xylocarpus granatum*; DMC, Diabetic Control mice; DMD, Diabetic mice+ Drug (Glibenclamide); DML, Diabetic mice + *Xylocarpus granatum* Low dose (100 mg/kg); DMH, Diabetic mice + *Xylocarpus granatum* High dose (200 mg/kg). ^a^p <0.05 compared with NC group. ^b^p<0.05 compared with DMC group.

Groups	Blood glucose level (mg/dl)				
	1^st^ Day	7^th^ Day	14^th^ Day	21^st^ Day	30^th^ day
NC	128.0 ± 6.32	131.6 ± 5.54	143.0 ± 9.13	151.2 ± 9.65	157.0 ± 9.77
NCT	125.4 ± 2.96	131.6 ± 3.91	144.4 ±13.59	149.0 ±14.86	154.0 ±15.76
DMC	311.4 ± 37.87^a^	346.2 ±30.93^a^	358.0 ±31.45^a^	380.6 ±37.36^a^	393.2 ±32.07^a^
		(+11.17%)	(+14.96%)	(+22.22%)	(+26.26%)
DMD	343.0 ± 56.43	316.6 ±56.93	285.6 ± 61.47	257.4± 42.67^b^	232.8±38.17^b^
		(-7.69%)	(-16.73%)	(-24.95%)	(-32.12%)
DML	322.2 ± 67.76	351.2 ± 72.59	338.6 ± 55.87	291.0 ± 61.15	261.2 ± 67.08
		(+9.0%)	(+5.09%)	(-9.68%)	(-18.93%)
DMH	324.0 ± 79.68	335.2 ± 64.37	308.4 ± 61.06	268.2± 40.73^b^	218.4±36.42^b^
		(+3.45%)	(-4.81%)	(-17.22%)	(-32.59%)

**Table 4:** Effect of *X. granatum *on serum biochemical parameters in STZ-induced diabetic mice. Data are expressed as mean ± SD, n=5. NC, Control mice; NCT, Normal Control mice +Toxicological (high) dose *Xylocarpus granatum*; DMC, Diabetic Control mice; DMD, Diabetic mice+ Drug (Glibenclamide); DML, Diabetic mice + *Xylocarpus granatum* Low dose (100 mg/kg); DMH, Diabetic mice + *Xylocarpus granatum* High dose (200 mg/kg). ^a^p< 0.05 compared with the control mice (NC); ^b^p< 0.05 compared with the diabetic control mice (DMC).

Groups	TG (mg/dL)	TC (mg/dL)	SGOT (U/L)	SGPT (U/L)	Urea(mg/dL)
NC	138.8 ± 5.89	102.6 ± 6.91	197.6 ± 13.84	89.6 ± 7.3	27 ± 1.58
NCT	152.6 ± 7.06^a^	87.8 ± 15.23	261 ± 19.72^a^	116.8 ± 8.95^a^	30.0 ± 1.0^a^
DMC	261.4 ± 11.43^a^	194.8 ± 12.3^a^	326.0 ± 9.38^a^	181.8 ± 8.01^a^	41.6 ± 2.60^a^
DMD	177.6 ± 6.23^b^	110.0 ± 17.42^b^	209.4 ± 25.48^b^	130.2 ± 5.21^b^	27.4 ± 2.07^b^
DML	219.4 ± 16.26^b^	131.6 ± 8.32^b^	242.6 ± 14.53^b^	152.4 ± 6.84^b^	35.2 ± 2.04^b^
DMH	187.2 ± 10.06^b^	104.6 ± 12.93^b^	198.6 ± 9.60^b^	118.6 ± 19.09^b^	28.4 ± 1.51^b^

**Table 5:** Effect of *X. granatum* on lipid peroxidation (LPx), non-protein-SH (NP-SH) and protein-SH (P-SH) in liver and brain tissues. Data are expressed as mean ± S.D. (n=5). NC, Control mice; NCT, Normal Control mice +Toxicological (high) dose *Xylocarpus granatum*; DMC, Diabetic Control mice; DMD, Diabetic mice+ Drug (Glibenclamide); DML, Diabetic mice + *Xylocarpus granatum* Low dose (100 mg/kg); DMH, Diabetic mice + *Xylocarpus granatum* High dose (200 mg/kg). ^a^p< 0.05 compared with the control mice (NC); ^b^p< 0.05 compared with the diabetic control mice (DMC).

Organs	Group	LPx	NP-SH	P-SH
		(nmolesTBARS/mg)	(*μ*M/g tissue)	(*μ*M/g tissue)
Liver	NC	0.60 ± 0.07	2.07 ± 0.28	4.91 ± 0.38
	NCT	0.54 ± 0.13	2.17 ± 0.20	4.38 ± 0.23
	DMC	0.75 ± 0.02^a^	2.18 ± 0.22	4.07 ± 0.23^a^
	DMD	0.77 ± 0.11	1.67 ± 0.37^b^	2.99 ± 0.15^b^
	DML	0.72 ± 0.19	1.81 ± 0.40	2.22 ± 0.33^b^
	DMH	0.73 ± 0.12	1.24 ± 0.19^b^	2.96 ± 0.48^b^
Brain	NC	1.68 ± 0.24	0.60 ± 0.07	4.34 ± 0.29
	NCT	0.66 ± 0.09^a^	0.56 ± 0.03	4.05 ± 0.21
	DMC	1.58 ± 0.27	0.61± 0.04	4.31 ± 0.43
	DMD	0.60 ±0.1^b^	0.59 ± 0.03	4.59 ± 0.29
	DML	1.22 ± 0.18^b^	0.55± 0.11	4.47 ± 0.20
	DMH	0.81 ± 0.17^b^	0.76 ± 0.07^b^	4.46± 0.77

**Table 6:** Effect of *X. granatum* on antioxidant enzymes in liver and brain tissues. Data are expressed as mean ± S.D., n=5. NC, Control mice; NCT, Normal Control mice +Toxicological (high) dose *Xylocarpus granatum*; DMC, Diabetic Control mice; DMD, Diabetic mice+ Drug (Glibenclamide); DML, Diabetic mice + *Xylocarpus granatum* Low dose (100 mg/kg); DMH, Diabetic mice + *Xylocarpus granatum* High dose (200 mg/kg). ^a^p< 0.05 compared with the control mice (NC); ^b^p< 0.05 compared with the diabetic control mice (DMC).

Organs	Group	SOD	CAT	GPx	GR	GST
		(U/mg)	(nKatal/mg)	(nmoles/min/mg protein)	(nmoles/min/mg protein)	(nmoles/min/mg protein)
Liver	NC	15.77 ± 2.80	3418.06 ± 375.19	26.11 ± 2.29	20.09 ± 4.92	2949.81 ± 116.54
	NCT	17.59 ± 4.20	2960.5 ± 761.71	30.62 ± 2.80^a^	20.69 ± 2.07	2416.51 ± 314.9^a^
	DMC	8.10 ± 0.40^a^	3297.6 ± 591.15	33.05 ± 1.31^a^	12.55 ± 1.48^a^	2083.95 ± 336.6^a^
	DMD	14.72 ± 1.42^b^	3792.69 ± 489.08	26.81 ± 0.82^b^	24.0 ± 1.84^b^	2093.61 ± 310.56
	DML	18.58 ± 4.13^b^	3892.74 ± 216.75	22.02 ± 2.71^b^	21.05 ± 2.23^b^	2416.51 ± 630.83
	DMH	18.35 ± 2.72^b^	4344.26 ± 779.38^b^	25.39 ± 1.11^b^	25.39 ± 3.28^b^	2016.54 ± 407.26
Brain	NC	12.87 ± 2.71	45.87 ± 13.44	26.42 ± 1.19	8.48 ± 0.43	261.64 ± 9.94
	NCT	12.02 ± 1.23	47.31 ± 11.01	30.22 ± 0.39^a^	9.8 ± 1.17^a^	306.22 ± 10.82^a^
	DMC	10.08 ± 0.39^a^	55.91 ± 7.44	33.19 ± 2.62^a^	6.32 ± 0.57^a^	222.89 ± 4.65^a^
	DMD	11.32 ± 2.4	37.27 ± 2.86^b^	18.08 ± 1.48^b^	11.81 ± 1.49^b^	270.81 ± 10.62^b^
	DML	10.56 ± 1.11	45.87 ± 9.93	23.27 ± 2.06^b^	7.32 ± 0.69	226.21 ± 6.84
	DMH	10.65 ± 1.92	41.57 ± 6.24	26.58 ± 1.14^b^	8.93 ± 0.69^b^	299.14 ± 8.0^b^

**Table 7:** Semi-quantitative scoring of histopathological examination of liver. (-): No change, (+): Positive for the parameter studied.

Tissue damage	NC	NCT	DMC	DMD	DML	DMH
Degeneration of hepatocytes	-	-	+	-	-	-
Fatty change in hepatocytes	-	-	+	+	-	-
Inflammatory cell infiltrations	-	-	+	+	-	-
Sinusoidal dilation	-	-	+	+	-	-

**Table 8:** Total flavonoid content and total tannin content of various extracts of *X. granatum*. The values are expressed as mean ± SD (n=3). EL = Ethanol leaf extracts; ML = Methanol leaf extracts; AL = Aqueous leaf extracts; EB = Ethanol Bark extracts; MB = Methanol Bark extracts; AB = Aqueous Bark extracts of *X. granatum*.

Sample	EL	ML	AL	EB	MB	AB
Total flavonoids	8.0 ± 0.20	8.0 ± 0.11	10 ± 0.09	7.0 ± 0.16	9.0 ± 0.15	8.0 ± 0.10
Total tannin	4.1 ± 0.03	5.54 ± 0.07	3.91 ± 0.09	9.76 ± 0.03	6.48 ± 0.04	5.28 ± 0.02

## Data Availability

The data used to support the findings of this study are included within the article.
